# Editorial: Insights and reviews in movement science 2023

**DOI:** 10.3389/fpsyg.2025.1706718

**Published:** 2025-10-20

**Authors:** Guy Cheron, Nadia Dominici, Susannah Williamson, Matthew Stults-Kolehmainen

**Affiliations:** ^1^Université Libre de Bruxelles, NeuroMove, ULB Neuroscience Institut (UNI), Brussels, Belgium; ^2^Department of Human Movement Sciences, Faculty of Behavioural and Movement Sciences, Vrije Universiteit Amsterdam, Amsterdam, Netherlands; ^3^I Corps, Joint Base Lewis-McChord, WA, United States; ^4^Sam Houston State University, Huntsville, TX, United States; ^5^Columbia University, New York, NY, United States

**Keywords:** motor control, movement, physical activity, sport, performance, psychosocial

## Introduction

This Research Topic encompasses three broad domains: (1) motor control, (2) fine-tuning incremental progress, and (3) psychosocial aspects of movement, physical activity, and exercise.

## Motor control: from normative data to innovative therapies

A unifying theme across this section is the quantification and enhancement of motor control through advanced assessment tools and innovative interventions that promote recovery and improve function. For example, in sports, elite marathons are one of the most impressive human endeavors. Yan et al. reviewed a large global database on the physiology, training intensity, pacing, nutritional strategies, age and sex differences, and recovery from inflammation and muscle damage during these events. By studying the relationships between the peak torque, peak power and mean power dynamometer output measures, Thompson re-analyzed the physiological implications of the isokinetic vs. isotonic operational principles largely utilized during sports training. The systematic review and meta-analysis by Julienne et al. provided the most comprehensive synthesis to date of normative data in instrumented posturography. Their findings underscore the influence of age and sex on postural control, while also exposing heterogeneity in protocols and the urgent need for standardization. Complementing this population-level view, Wang et al. examined how neuromuscular coordination is reorganized under fatigue conditions. Using EMG coupling analysis during bench press exercises, the authors revealed altered synchronization between agonist and antagonist muscle pairs, pointing to supraspinal adaptations that compensate for declining muscle force. This mechanistic insight advances our understanding of how fatigue reshapes motor output and informs both athletic training and rehabilitation practices. Czyż et al. approached the theme of interventions from a motor learning perspective by reviewing nearly six decades of research on contextual interference. Their systematic review and meta-analysis confirmed that random practice schedules enhance transfer, with a medium effect size overall, especially among adults and older adults. The introduction of virtual reality (VR) environments into the field of physical activity opens new avenues for exploring performance enhancement, therapeutic interventions, and health promotion. Interestingly, the review of Yu et al. confirmed that a single session of transcranial direct current stimulation (tDCS) has the potential to improve motor performance in healthy subjects and athletes. In their 2024 review, Schedler et al. provided an overview of recent advances in high-resolution head-mounted displays (HMDs) and their application in training balance-related skills across the lifespan. The use of HMDs should therefore benefit the subject's overall balance, particularly during the critical phases of maturation. In the same line of inquiry, Xue et al. addressed how to leverage technology to enhance motor outcomes in children with cerebral palsy. Their systematic review and meta-analysis demonstrated that virtual reality motor games significantly improve both gross and fine motor skills. The integration of molecular genetic technologies, epidemiology, exercise physiology and biostatistics to investigate quantitative performance traits, such as muscle power output and movement recording with new motion capture was reviewed by Papadimitriou. This study focused on the implications for the biotech industry, particularly in gene therapy to combat age-related muscle power decline and personalized medicine, which will drive advancements in exercise program design. Zi and de Geus re-analyzed the genetic aspect of the Stodden model regarding the potential confounding factors of familial environment characteristics, which include household, neighborhood, and parental rearing style. Collectively, these contributions advance our understanding of motor control mechanisms, from biomechanics to genetics, and highlight promising avenues for both athletic training and rehabilitation.

## Incremental progress and “fine-tuning”

Recent works have promulgated the idea of fine-tuning in exercise, physical activity and sports. This process implies incremental progress in motor development, athletic training programs, and scientific research through tweaking and slight adjustments.

Nowhere is skill development more essential than in the transition from youth to adult athlete. The study by Zheng and van der Kamp considered the strengths and limitations of the body-scaled approach often utilized in youth sports. It was highlighted that an action-scaled approach, which emphasizes task specificity, helps better guide youth sports modifications while effectively developing young athletes for the next stage of their careers.

Balance training is a particularly valuable skill for both individual athletes and teams. Chen et al. found that balance training, when combined with multi-directional movement exercises, improved the ability to change direction more quickly among young table tennis players. Similarly, the review by Gao et al. focused on the effects of instability resistance training on athletes' performance. This type of training has proven effective for improving dynamic balance abilities and core stability among many strength, power, and even endurance athletes.

The ability to monitor brain activity during athletic performance has improved significantly over the past several years. A systematic review of functional near-infrared spectroscopy (fNIRS) by Shen et al. showed that physical exercise induces positive adaptations in both the prefrontal and motor cortices through increased levels of oxygenated hemoglobin. These changes in cortical hemodynamics were found to be associated with enhanced inhibitory control and working memory. Another study by Yu et al. examined the effects of a single session of transcranial direct current stimulation (tDCS) and found significant improvements in strength, endurance, and emotional state, with smaller but still notable effects on sport-specific tasks and cognitive performance.

Implications for improved cognitive performance were also highlighted by a systematic review on eye-tracking technology (Kredel et al.). While progress in this field has been uneven since the last update, one important advancement is the use of automated gaze-cue allocations (GCAs), particularly in mobile eye-tracking studies. GCAs increase objectivity while reducing the manual workload.

Taken together, these findings reinforce the importance of fine-tuning, highlight promising avenues for future research, and suggest further applications aimed at enhancing athletic performance and excellence.

## Psychosocial aspects of movement, physical activity, and exercise

Five contributions (Segar; Carrera-Bastos et al.; Jadhakhan et al.; Yang et al.; Si et al.) were specifically related to the psychosocial aspects of physical activity (PA) and exercise. One could consider motivation to be the predominant theme of these works. At first glance, tying these papers together into a single motivational model may seem unwieldy. However, conceptual and theoretical advances have made this feasible. Taken together, these studies propose that an immediate antecedent of movement is an urge or impulse to move or not move. These articles also include important fundamental aspects of motivation, such as active approach vs. active withdrawal from physical activity and sedentary behaviors, along with negative reinforcement and classic aspects of drive theory. In addition, there is a likely motivational null point where both approach and withdrawal are neutralized and wants/desires are minimized, a state similar to mindfulness, as described by Si et al.. Potentially dozens of factors have an interplay in these processes, but in terms of approach motivation, the article by Segar suggested that promoting PA/exercise over the long term could be accomplished by: 1) enhancing the affective response to PA, 2) challenging all-or-nothing decision-making, and 3) giving oneself permission for self-care, such as structured exercise.

Enhancing motivation for activity in healthy populations is quite different from minimizing pathological processes that inhibit movement. In the case of depression, withdrawal (or sometimes excessive activity) is a common hallmark of the disorder, known as psychomotor retardation or excitation. This is potentially related to dysregulated inflammatory processes (Carrera-Bastos et al.) which can be counteracted by a variety of different exercise interventions. Fear of movement (i.e., kinesiophobia), as explained by Jadhakhan et al., is an example of active withdrawal, dread, or aversion to movement, a state particularly activated by the experience or anticipation of pain. While it is understood that the majority of individuals suffer from too little movement, some subjects move too much, to the extent that their movement tendencies interfere with other objectives in their lives. With ADHD, it also seems likely that there are excessive urges to move (Yang et al.) and this should be studied more systematically in the future. Paralympic sports such as Boccia also demonstrate that, beyond their physical benefits, they emphasize social interaction, emotional regulation and empathy (Ferreira et al.). These findings illustrate the complex interplay between psychological, biological, and social factors in sustaining healthy movement behaviors.

